# Family-based genetic risk prediction of multifactorial disease

**DOI:** 10.1186/gm123

**Published:** 2010-01-15

**Authors:** Douglas M Ruderfer, Joshua Korn, Shaun M Purcell

**Affiliations:** 1 Psychiatric and Neurodevelopmental Genetics Unit, Center for Human Genetic Research, Mass General Hospital, Boston, MA, USA; 2 The Stanley Center for Psychiatric Research, The Broad Institute of Harvard and MIT, Cambridge, MA, USA; 3 Broad Institute of Harvard and MIT, Cambridge, MA, USA; 4 Department of Psychiatry, Harvard Medical School, Boston, MA, USA

## Abstract

Genome-wide association studies have detected dozens of variants underlying complex diseases, although it is uncertain how often these discoveries will translate into clinically useful predictors. Here, to improve genetic risk prediction, we consider including phenotypic and genotypic information from related individuals. We develop and evaluate a family-based liability-threshold prediction model and apply it to a simulation of known Crohn's disease risk variants. We show that genotypes of a relative of known phenotype can be informative for an individual's disease risk, over and above the same locus genotyped in the individual. This approach can lead to better-calibrated estimates of disease risk, although the overall benefit for prediction is typically only very modest.

## Background

Although whole-genome association studies have detected dozens of common variants for a broad range of complex diseases, and are likely to detect many more, the total variance explained by the known variants is typically modest [1,2]. As such, realising the goals of accurate genetic risk prediction and the subsequent opportunities of personalised medicine remains difficult [3,4]. Indeed, it has often been noted that family history alone will perform substantially better as a predictor of risk, compared to genotype data for known risk variants [5]. It is true that a positive family history will likely remain an important factor in prediction for the many complex diseases with substantial heritabilties and shared familial environmental components. (A caveat is that family history information might sometimes not be straightforwardly available, for example, for phenotypes such as response to a particular drug treatment.) However, analogous to clinical genetic testing for Mendelian disease, it is plausible that in many cases a positive family history will itself be a motivating factor for pursuing a genetic test. For example, an individual whose older sibling developed a particular disease might be particularly concerned with their own personal risk, which they assume will be higher than average. In this context, in which a genetic test is sought because a first-degree relative has disease, we developed a family-based model for risk prediction incorporating genotype data from both the index individual and a relative of known phenotype. As such, we do not ask "how well do SNPs predict disease compared to family history", but rather, "how well do SNPs predict disease given a positive family history, and to what extent does including genotype data from the affected relatives help?".

### Information from relatives of known phenotype

For diseases with polygenic and shared environmental components of risk, the genotype of a relative of known phenotype can be informative for an individual's disease risk, over and above the individual's own genotype at that locus. Below, the term genotype here refers to both single and multi-locus genotypes, unless explicitly stated. We assume that genotypes at the locus or loci under consideration only account for a proportion of the total familial covariance, meaning that unmeasured residual polygenic and/or shared environmental factors still exist, as would be expected for a complex disease.

Ignoring the relative's phenotype, then as expected, in an unselected population a relative's genotype does not predict the index individual's disease risk given the index's own genotype. That is, if index disease *D*_*I *_is modeled as a function of index genotype *G*_*I *_and, for example, sibling genotype *G*_*S*_

then *E*(*b*_2_) = 0 even if *E*(*b*_1_) ≠ 0. However, if we know the phenotype of the sibling, *D*_*S*_, and include it in the model

then if *E*(*b*_1_) > 0, for example, *E*(*b*_2_) will no longer equal zero. In fact, in this case, *E*(*b*_2_) < 0 meaning that the sibling's genotype is informative for the index's disease risk, in the opposite direction compared to *b*_1_.

Why is the sibling genotype conditional on index genotype and sibling phenotype informative for index disease risk? For a given risk locus, if the sibling is affected but has a low-risk genotype, this implies that the index is at *higher *risk than if the affected sibling has a high-risk genotype, conditional on the index's own genotype at that locus. In this scenario, the affected sibling's genotype acts as a surrogate for all other *unmeasured *risk factors: if the sibling has the low-risk genotype but still is affected, he or she is likely to have a higher rate of other, unobserved risk factors, either genetic or environmental. To the extent that these unobserved risk factors are shared among siblings, the affected sibling's genotype will therefore act as a surrogate for the index's unobserved risks. This is analogous to the epidemiological phenomenon of selection bias, in which an association arises due to shared but unmeasured factors.

In general, a lower genetic load of known risk variants in an affected relative will tend to increase the index's risk of disease, over and above the level of risk predicted by the index's own genotype. For the index, a higher genetic load still leads, as usual, to a higher predicted risk. (Note that if we did not know the index genotype, the affected relative's genotype would act as a surrogate for it. In this case, a higher load of known risk variants in the affected relative would predict a higher, not lower, risk in the index. Unless the affected relative is an MZ twin, prediction would naturally be worse than if we knew the actual index genotype.) In the rest of this report, we applied this observation to the problem of genetic risk prediction, asking whether the inclusion of genotypes from a relative of known phenotype can improve the accuracy of prediction.

## Methods

### Prediction model incorporating family information

Here we introduce a model in which the relative of known phenotype is an affected sibling; the basic approach can be easily extended to other and multiple relative types. Specifically, we wish to predict disease risk for the index individual, conditional on a) their multilocus genotype at *V *known disease variants, b) their affected sibling's disease state and c) additionally including the affected sibling's multilocus genotype.

For two siblings (with subscripts *I *and *S *for the index and affected sibling, respectively), we model disease state *D *given genotypes *G *at one or more loci. Estimates of population allele frequencies and relative risks for *G *are assumed to be known in advance. The probability that the index develops disease given both their and their affected sibling's genotype at a single locus is

where *P*(*D*_*I*_, *D*_*S*_|*G*_*I*_, *G*_*S*_) and  are directly obtained from the multivariate normal cumulative distribution function, assuming a liability-threshold model for disease risk.

The liability-threshold model assumes an unobserved, normally-distributed liability (*Q*); individuals with liability values above a threshold are affected. For threshold *t*, *P*(*Q *≥ *t*) = *k *where *k *is the specified population prevalence of disease. For two family members, the probability of joint sibling disease state *D *given genotypes *G *is

and the joint cumulative distribution of *Q *is given by the multivariate normal distribution function

The expected value of *Q *is a function of the genotypes for each sibling, *G*_*I *_and *G*_*S*_; the residual variance is partitioned into the components of variance representing polygenes (), family-wide common environmental factors () and individual-specific, or nonshared, factors, including measurement error (). These variance components must be specified in advance, for example, from twin and family studies. For a given individual, we use the likelihood ratio as a measure of risk of being affected, *D*_*I*_, versus unaffected, [6], extended here to incorporate genotypic and phenotypic information on the sibling, *G*_*S *_and *D*_*S*_,

where

and *P*(*G*_*I*_, *G*_*S*_, *D*_*S*_) = *P*(*G*_*I*_, *G*_*S*_|*D*_*S*_)*P*(*D*_*S*_). The population joint sibship genotype frequencies *P*(*G*_*I*_, *G*_*S*_) are calculated assuming random mating and Hardy-Weinberg equilibrium in the population, summing over all possible parental mating and transmission types. Conditioning on proband disease state, then

These likelihoods can be combined across multiple independent loci, as log(*L*_*M*_) = ∑_*v*_log(*L*_*v*_) where *L*_*v *_is the likelihood ratio for variant *v*. Then, following Yang et al. [6], the risk of disease for the index is given by

### Simulation study of Crohn's disease variants

We simulated data to approximate the set of 30 risk variants reported in Barrett et al as follows. We set the disease prevalence to *k *= 1/250. (In practice, determination of affection status was based on fixed threshold on the normal liability scale, and so the implied prevalence will vary slightly around 1/250 when non-null genetic effects are specified. This effect is very small and does not impact the comparisons of methods and conclusions, however.) The risk allele frequency (RAF) and genotypic relative risk (GRR) for each variant are reported in Table 1. Given *k*, RAF and GRR for each variant, we estimated the implied additive genetic value *a *by numerical optimization.

**Table 1:** Crohn's disease model specification

RAF	GRR	*a*	VE
0.018	3.99	0.504	.0090
0.533	1.28	0.098	.0048
0.425	1.25	0.083	.0034
0.899	1.31	0.135	.0033
0.387	1.25	0.083	.0032
0.152	1.35	0.106	.0029
0.677	1.22	0.080	.0028
0.463	1.21	0.071	.0025
0.478	1.20	0.067	.0023
0.678	1.20	0.072	.0022
0.780	1.21	0.079	.0022
0.221	1.25	0.079	.0022
0.933	2.50	0.130	.0021
0.125	1.32	0.097	.0021
0.565	1.18	0.062	.0019
0.565	1.18	0.062	.0019
0.697	1.18	0.064	.0017
0.271	1.20	0.065	.0016
0.090	1.33	0.099	.0016
0.243	1.19	0.061	.0014
0.386	1.16	0.053	.0013
0.289	1.17	0.055	.0013
0.345	1.16	0.053	.0013
0.682	1.14	0.049	.0010
0.389	1.13	0.043	.0009
0.473	1.12	0.040	.0008
0.348	1.12	0.040	.0007
0.017	1.54	0.149	.0007
0.708	1.11	0.038	.0006
0.619	1.08	0.027	.0004

Values used to generate simulated CD samples. RAF = risk allele frequency; GRR = genotypic relative risk, estimated from the reported odds ratios; *a *= additive genetic value; VE = variance explained.

In all cases, we set the polygenic variance components  = 0.7,  = 0.2 and  = 0.1, which implies a risk to individuals with at least one affected sibling of 0.11, and therefore, a sibling relative risk of 28.6 [7]. Note that the performance of the family model depends on the residual sibling correlation  and not just the individual values of values of  and  (i.e. all pairs of values that yield the same implied sibling correlation will show identical performance).

For the unselected population we simulated 500,000 nuclear families, each with two siblings. For the family-history positive population, we simulated 100,000. Fewer replicates were required due to the much higher baseline rate for *D*_*I *_in this population.

## Results and discussion

### Single locus example

**Figure 1:**
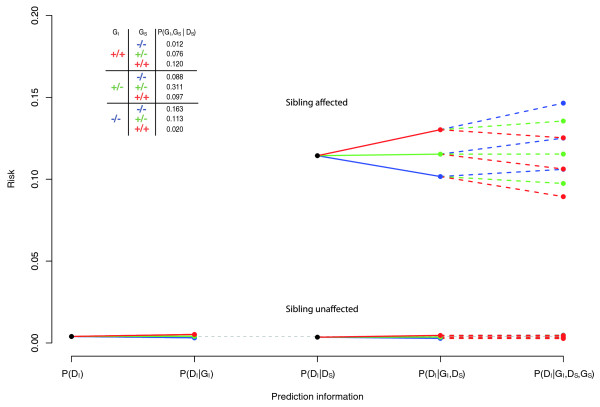
**Predicted index disease risk**. Predicted index disease risks from a single locus (MAF = 0.425, GRR = 1.25): unconditonal, *P*(*D*_*I*_); conditional on index genotype, *P*(*D*_*I*_|*G*_*I*_); conditional on affected sibling phenotype, *P*(*D*_*I*_|*D*_*S*_); conditional on index genotype and affected sibling phenotype, *P*(*D*_*I*_|*G*_*I*_, *D*_*S*_); conditional on index and sibling genotypes and affected sibling phenotype, *P*(*D*_*I*_|*G*_*I*_, *G*_*S*_, *D*_*S*_). The inserted table contains frequencies of sibling pair genotype combinations conditional on at least one sibling being affected. Red represents the homozygous risk-increasing genotype; green the heterozygous genotype; blue the homozygous risk-decreasing genotype.

To illustrate the approach, we analytically calculated the expected risk under a variety of models, based on information from a single locus - rs2188962, one of the Crohn's disease (CD) loci identified in a recent meta-analysis [8], setting the genotypic relative risk (GRR) to 1.25 and the risk allele frequency (RAF) to 0.425. Prevalence, additive polygenic and shared environmental components of variance were set to approximate known values for CD, as described above. Figure 1 shows the predicted disease risks under five models:

no information, *P*(*D*_*I*_);

conditional on index genotype, *P*(*D*_*I*_|*G*_*I*_);

conditional on having an affected sibling status alone, *P*(*D*_*I*_|*D*_*S*_);

as above, including index genotype, *P*(*D*_*I*_|*G*_*I*_, *D*_*S*_);

as above, including sibling genotype, *P*(*D*_*I*_|*G*_*I*_, *G*_*S*_, *D*_*S*_).

Conditional on index genotype, the affected sibling's genotype further stratifies risk, but with the low-risk genotype predicting increased risk for the index. Values of *P*(*D*_*I*_|*G*_*I*_) only range around *P*(*D*_*I*_), from 0.32% to 0.52% for the low-risk to high-risk homozygotes, whereas *P*(*D*_*I*_|*G*_*I*_, *G*_*S*_, *D*_*S*_) shows a much greater range around P(*D*_*I*_|*D*_*S*_), from 8.9% to 14.6%. The predicted risks shown here were reproduced by simulating data under this model and calculating the proportion of index cases for each configuration (data not shown).

**Figure 2:**
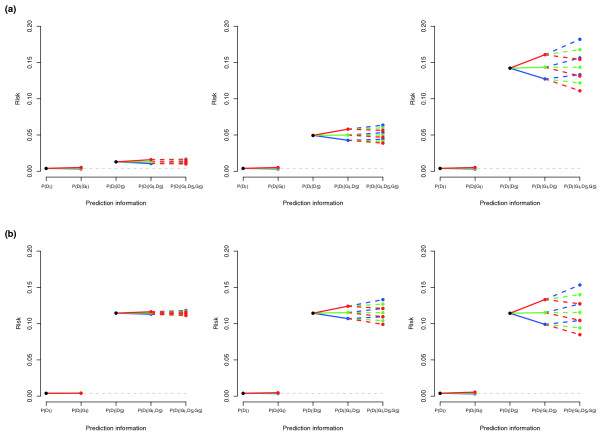
**Predicted index disease risks from a single locus, under a variety of genetic models.** Predicted index disease risk stratified by **(a)** effect size and **(b)** total sibling relative risk. See Figure 1 legend for details. In all cases, risk allele frequency is 0.425, disease prevalence is 1/250. (a) Varying the familial variance component of the residual variance from 20%, 50% to 80%, with corresponding sibling relative risks of 3.25, 12.25 and 35.5. (b) Varying additive genetic effect from *a *= 0.01, *a *= 0.05 to *a *= 0.1, with corresponding genotypic relative risks of 1.03, 1.16 and 1.30.

Figure 2 illustrates the relative performance of the different models under varying levels of effect size and background residual familial variance. In general, the absolute and relative impact of the affected sibling's genotype increases with both of these factors.

### Crohn's disease simulation

We next performed a simulation as described above that included all 30 CD variants reported in Barrett et al [8] that collectively account for 6.4% of the total variance (calculated assuming a liability-threshold model and assuming additivity across loci on the scale of liability). We first simulated a simple unascertained sample of nuclear families, each with two siblings (i.e. *D*_*S *_will only be affected at the usual population prevalence). Second, we used rejection sampling to simulate an ascertained sample in which at least one sibling was affected (*D*_*S *_is always affected). For each simulated family, we calculated the risk for the index being affected, *D*_*I *_using the methods described above.

We evaluated performance using three metrics: 1) the area under ROC curve (AUC), 2) the squared correlation between true disease state and predicted risk (*R*^2^), and 3) the enrichment in the rate of cases versus the population prevalence for individuals in the highest 1, 5, or 10% of estimated risk (*T*_1_, *T*_5 _and *T*_10_). We assessed performance for three models: *P*(*D*_*I*_|*G*_*I*_), *P*(*D*_*I*_|*G*_*I*_, *D*_*S*_) and *P*(*D*_*I*_|*G*_*I*_, *G*_*S*_, *D*_*S*_). All results are shown in Table 2.

**Table 2:** Crohn's disease simulation results

Model	AUC	*R* ^2^	*T*_1_	*T*_5_	*T*_10_
General population	0.708	0.054	7.39	4.21	3.23
*P*(*D*_*I*_|*G*_*I*_)	0.726	0.085	15.90	5.71	3.91
*P*(*D*_*I*_|*G*_*I*_, *D*_*S*_)	0.735	0.094	15.88	5.80	3.94
*P*(*D*_*I*_|*G*_*I*_, *G*_*S*_, *D*_*S*_)					
					
Selected population (affected sibling)					
*P*(*D*_*I*_|*G*_*I*_, *D*_*S*_)	0.628	0.042	71.25	60.25	53.75
*P*(*D*_*I*_|*G*_*I*_, *G*_*S*_, *D*_*S*_)	0.648	0.056	82.00	67.20	58.48

Performance characteristics for tests based on the 30 Crohn's disease variants. Index individuals and their siblings were simulated in the unselected and selected (family history positive/affected sibling) scenarios. The prediction models estimate risk based on the index genotype *G*_*I*_, and optionally sibling's phenotype *D*_*S *_and genotype *G*_*S*_. The metrics are the area under the ROC curve (AUC), the squared correlation between disease state and risk (*R*^2^) and the relative enrichment of cases in the top 1, 5 and 10% of individuals with the highest risk scores relative to the baseline risk for that population (*T*_1_, *T*_5 _and *T*_10_). See main text for details.

We first describe results for the general population, in which nuclear families were generated without any ascertainment on disease. As expected, compared to the basic model *P*(*D*_*I*_|*G*_*I*_), the inclusion of a sibling phenotype *D*_*S *_(which might be affected or unaffected) improved both risk prediction for the index, particularly as indexed by *R*^2 ^(0.054 to 0.085). The enrichment of cases in the highest-ranked 1% (*T*_1_) more than doubled (7.39 to 15.9). In this population, however the addition of the sibling's genotypes *G*_*S *_added only marginal benefit in terms of AUC and *R*^2^, and no benefit for the *T *metrics.

In the second population, we ascertained for a positive family history (i.e. *D*_*S *_is always affected). Of note, compared to the unselected population, the AUC and *R*^2 ^metrics are considerably lower in this high-risk population, whereas the *T *metrics are substantially higher (largely reflecting the high sibling relative risk for this disease). That the discriminative performance of a test may vary depending on the characteristics of the population it is deployed in may have important implications for the generalizability of studies that claim a certain AUC, which is not an invariant property of the test alone but depends on the context in which it is used.

In terms of discrimination, the basic *P*(*D*_*I*_|*G*_*I*_) model as expected yields near identical results compared to *P*(*D*_*I*_|*G*_*I*_, *D*_*S*_), as all siblings are affected in this population; we therefore omit this model here. However, the absolute values of predicted risk based on *P*(*D*_*I*_|*G*_*I*_) will be very poorly calibrated, as this model ignores the presence of a positive family history. For example, for individuals with a predicted risk of 0.1 ± 0.01 from the *P*(*D*_*I*_|*G*_*I*_, *D*_*S*_) model, we observed a rate of 0.099 cases in the simulated data. However, based on *P*(*D*_*I*_|*G*_*I*_), these same individuals had a mean predicted risk of only 0.0037. In other words, by not conditioning on known affected sibling status, the prediction model will dramatically under-estimate the absolute risks.

Finally, we considered whether adding sibling genotypes improved prediction in this family-history positive population. We observed negligible improvement in AUC (1.03-fold increase) but a larger increase for *R*^2 ^(1.33-fold, 0.042 to 0.056). There were also increases in the already-large *T *metrics. As expected, the benefit derived from including sibling genotypes is larger in the ascertained population, as for a relatively rare but highly familial disease, affected siblings will be more informative than unaffected siblings. In the family-history positive population, adding affected sibling genotypes offers some advantage, although likely not enough to ever fundamentally change the discriminative utility of a test.

Including affected sibling genotypes can improve the calibration of predicted risks somewhat and lead to a greater stratification of risk, as apparent in Figure 1. We can quantify the risk stratification depicted in Figure 1 in terms of a metric *δ*. Comparing two sets of predicted risks, we define *δ *as the expected change in risk, calculated as ∑_*i*_|*P*_*i*_-*Q*_*i*_|/*N *of *N *total individuals, *P*_*i *_is the probability of disease in the individual before the test and *Q*_*i *_is the probability afterwards. This is one way of characterizing the personal impact of a test: the expected change in estimated risk pre- versus post-test. In the family-history positive population, *δ *for *P*(*D*_*I*_|*G*_*I*_, *D*_*S*_) is 0.035; the incremental *δ *going from the risks estimated based on *P*(*D*_*I*_|*G*_*I*_, *D*_*S*_) to *P*(*D*_*I*_|*G*_*I*_, *G*_*S*_, *D*_*S*_) is 0.02. In other words, updating one's risk based on an affected sibling's genotype would be expected to change one's predicted risk 57% (0.02/0.035) as much as the initial test (in the unselected population, this value is 50%).

### Including additional and/or unaffected family members

We also considered models in which additional affected family members are included in the model: for example, individuals in multiplex families with an affected sibling and an affected parent, or two affected siblings. In general, we do see improvement from incorporating the genotypes of these additional affected relatives, although there tends to be a diminishing return (data not shown).

In practice, for most diseases, being of relatively low frequency (e.g. under 10%), only affected relatives will contribute information, compared to relatives known to be disease-free. In addition, determination that an individual is disease-free with respect to life-time risk might be difficult.

### Limitations

One caveat is that if the known variants used in the test themselves account for the entire familial covariance, then genotypes from phenotyped relatives will not contribute any additional information. This is unlikely to be the case in the foreseeable future for most diseases, however; it would imply that we have already maximized the potential of genetic risk prediction.

For this work we have assumed a particular model for risk, additivity on the scale of liability, which in practice approximates a multiplicative model on the scale of risk. This implies that the same risk ratio will correspond to a larger absolute risk difference if there is a higher baseline risk: for example, 1% versus 2% and 5% versus 10% both imply risk ratios of 2, but varying absolute risk differences. This effect is evident in Figure 1, in which genotype leads to a greater stratification of absolute risk in individuals with an affected sibling. Whether or not the implied penetrances for individuals with a positive family history actually follow this model is a question that ultimately should be empirically addressed, to indicate the adequacy of the risk model. However, this does not alter the qualitative principle outlined here that relatives' genotypes and phenotypes are informative for an individual's disease risk.

## Conclusions

We observed that the genotypes of relatives of known phenotype are informative for an individual's risk, independent of the same risk variants measured in the index individual. We sought to determine whether this phenomenon could be of use in the context of genetic disease risk prediction. We described and evaluated a prediction model for individuals with one or more affected first-degree relatives. Our model has the key feature of incorporating genotype information from relatives to improve the accuracy of prediction. The basic insight - that affected relatives' genotypes are informative about an individual's risk for a multifactorial, polygenic disease - is not confined to the particular analytic approach presented here and could be used with other prediction methodologies. In this work, we focused on the additive effects of confirmed disease alleles, although others have incorporated other sources of information, including non-genetic risk factors [9] and interactions between risk factors [6]. To the extent that such risk factors are shared between relatives, the approach outlined here to include information from affected relatives could also be applied in these other contexts. Methodologically, we used a liability threshold model. Others have developed prediction models using logistic regression [6], optimal ROC curves [10], Bayesian networks [11] and support vector machines [12], using diverse criteria to evaluate performance in terms of, for example, discrimination, calibration and reclassification [13]. Again, information from affected relatives could in theory be included using any of these approaches. In fact, our approach is conceptually similar to methods in livestock genetics and animal breeding that use genetic marker data for prediction, using all the data and taking into account familial relationships in complex pedigrees [14]. However, in the context of human disease risk prediction, our simulations suggest that, in most cases, only incremental improvements are to be expected, meaning it is unlikely that the overall applicability of a test will be fundamentally altered.

## Abbreviations

AUC: area under the curve; CD: Crohn's disease; GRR: genotypic relative risk; MAF: minor allele frequency; MZ: monozygotic; RAF: risk allele frequency; ROC: receiver operating characteristic; SNP: single nucleotide polymorphism; VE: variance explained.

## Competing interests

The authors declare that they have no competing interests.

## Authors' contributions

All authors contributed to the conception of this project. SMP and DMR developed and implemented the methods. DMR and SMP designed and performed the simulations. All authors contributed to the drafting of the manuscript.
